# Healthcare resource utilization, total costs, and comorbidities among patients with myotonic dystrophy using U.S. insurance claims data from 2012 to 2019

**DOI:** 10.1186/s13023-022-02241-9

**Published:** 2022-02-23

**Authors:** Sarah J. Howe, David Ladipus, Michael Hull, Jason Yeaw, Tanya Stevenson, Jacinda B. Sampson

**Affiliations:** 1 Marigold Foundation, 7515 Flint Road SE, Calgary, AB T2H 1G3 Canada; 2 LapidusData Inc., Oklahoma City, OK USA; 3 IQVIA, Falls Church, VA USA; 4 Myotonic Dystrophy Foundation, Oakland, CA USA; 5 Stanford University, Stanford, CA USA

**Keywords:** Myotonic dystrophy (DM), Healthcare resource utilization (HCRU), Insurance claims database, Dystrophy, Rare disease, Congenital myotonic dystrophy, Myotonic dystrophy type 1, Myotonic dystrophy type 2, Healthcare costs, Dystrophia myotonica

## Abstract

**Background:**

Myotonic dystrophy (DM) is a rare, inherited disorder with multi-systemic effects that impact the skeletal muscles, eyes, heart, skin and gastrointestinal, endocrine, respiratory, and central nervous systems. DM is divided into two subtypes: DM1 can present from early childhood through adulthood and also has a congenital form (cDM) while DM2 typically manifests during mid-adulthood. Both forms are progressive with no approved treatments, and unmet need for disease-modifying therapies remains high. This study interrogated health insurance claims data to explore the clinical experience, healthcare resource utilization (HCRU), and all-cause costs for DM.

**Results:**

A total of 8541 patients with DM and 242 patients with cDM and their matched controls were selected from a database of over 200 million claimants. HCRU and all-cause costs, including pharmacy, outpatient, and inpatient services, were analyzed across four years in 12-month follow-up periods. Mean all-cause costs per DM patient were high in each of the four periods (range $14,640–$16,704) and showed a steady increase from 13 to 23 months on, while the control group mean costs declined from $9671 in the first 12 months after the index event, to approach the US population average ($5193) over time. For cDM, the highest mean costs were in the first 12-months ($66,496 vs. $2818 for controls), and remained high (above $17,944) across all subsequent periods, while control mean costs approached $0. For DM and cDM, HCRU was higher compared to controls across all study periods and all-cause healthcare costs were mostly driven by inpatient and outpatient encounters. Analysis of all diagnosis codes over the study period (comorbidities) demonstrated an elevated comorbidity profile consistent with the clinical profile of DM.

**Conclusions:**

This study is among the first to utilize claims data to increase understanding of the clinical experience and health economic outcomes associated with DM. The markedly elevated HCRU patterns and comorbidity profile presented here add to the broad body of scientific and clinical knowledge on DM. These insights can inform clinical care and support the development of disease modifying and/or symptom-targeting therapies that address the multi-systemic, progressive nature of DM.

**Supplementary Information:**

The online version contains supplementary material available at 10.1186/s13023-022-02241-9.

## Introduction

Myotonic dystrophy (DM) is a complex, multi-systemic neuromuscular disease that is characterized by delayed relaxation of skeletal muscles (myotonia) and progressive muscle weakness and degeneration (muscular dystrophy) [1–4]. It is a rare and highly variable disease, affecting numerous parts of the body, including skeletal muscle, eyes, heart, and gastrointestinal (GI), endocrine, CNS, skin, and respiratory systems [2, 5–7]. DM is an inherited disorder that is divided into two subtypes, myotonic dystrophy type 1 (DM1) and myotonic dystrophy type 2 (DM2), both of which result from polynucleotide repeat expansions [8, 9]. DM as a whole represents the most common muscular dystrophy in adults. DM1 can present from early childhood through adulthood and has a congenital form (cDM) while DM2 typically manifests during mid-adulthood [10–13]. Adult patients with DM present with variable signs and symptoms, including muscle weakness and atrophy, fatigue, cardiac abnormalities, sleep disorders, and CNS, GI tract, or endocrine dysfunctions, among others [4]. Patients with cDM may present with neonatal hypotonia, dysphagia, respiratory failure, and cognitive impairments [14]. Although no approved treatments exist for DM, recent advances in therapeutic drug development are promising [15–17].

While past research has demonstrated a high disease burden for patients with DM, the healthcare resource utilization (HCRU) and healthcare costs for this understudied population are relatively unexplored [3, 18, 19]. This type of data is needed to help inform scientific research, clinical care practices, advocacy organizations, regulatory and government agencies (e.g., NIH, CDC, FDA), as well as therapeutic drug development and discovery efforts (i.e., academic and industry). Using health insurance claims data, this study sought to provide direct evidence of HCRU and total all-cause healthcare costs among patients with DM across several years. Analysis of these data provides new clinical and economic insight into the DM experience for the benefit of patients, caregivers, and payers.

## Methods

This retrospective, observational, real-world study was conducted using claims data derived from the IQVIA New Data Warehouse, which consists of the Professional Fee Claims database (outpatient claims from physician and specialist offices) with linkage to the Prescription Claims database (linking applies a deterministic matching algorithm using patient information to ensure continuity of patient records across datasets). The IQVIA Hospital Charge Data Master database was used for certain patient outcomes, such as inpatient stays. The IQVIA New Data Warehouse extracts de-identified data from non-federal community acute-care short-stay hospitals, providers, and pharmacies. It includes records from more than 450 hospitals, clinical data representing 60%**–**70% of US-based physician activity, and approximately 85% of dispensed prescriptions for all pharmacies in the US. Similar methods have been used to characterize the HCRU and costs associated with medical management of spinal muscular atrophy (SMA), ankylosing spondylitis, and Duchenne muscular dystrophy (DMD) [20–22].

**Fig. 1:**
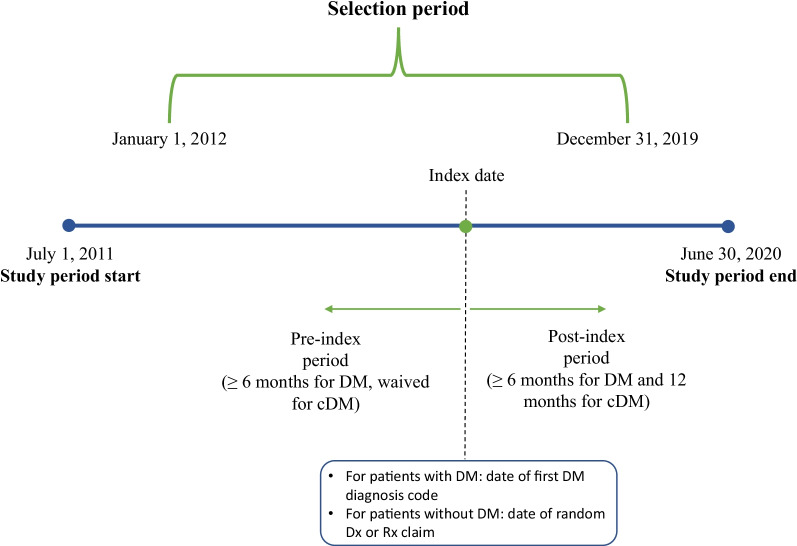
Patient selection and study period

This study spanned from July 1, 2011 through June 30, 2020 (study period; Fig. 1). DM patients were identified between January 1, 2012 through December 31, 2019 (selection period) based on index date, which was the date of the first of two observed International Classification of Diseases (ICD) diagnosis codes for DM (the ICD-9 code for DM is 359.21 and the ICD-10 code [introduced after October 2015] is G71.11). The ICD-9 and ICD-10 codes do not differentiate between DM1 and DM2, nor cDM and DM.

In an effort to investigate the unique congenital myotonic dystrophy experience, a separate subcohort was identified using a criterion of < 2 years of age at index date as a surrogate for a diagnosis of cDM. This early window was imposed to capture those study participants who received a diagnosis code for DM within the first two years of life and who, therefore, are most likely representative of the early childhood cDM experience. All other study participants remained in the DM cohort, which includes DM1 and DM2 patients 2 years of age and older at index date.

For the DM cohort, the required pre-index (baseline) period was 6 months prior to the index date; however, this period was waived for inclusion in the cDM cohort. The index period is considered to be pre-diagnostic for DM patients. The variable post-index (follow-up) period was a minimum of 6 months for DM and 12 months for cDM, beginning on the index date. Continuous patient eligibility in the pre- and post-index periods (post-index only for the cDM cohort) was required. Six months of pre- and post-index pharmacy stability was also required for the DM cohort. This requirement was adjusted to only 12-months post-index for the cDM cohort, as many of these patients receive prescription medications outside the pharmacy system as part of neonatal intensive care.

The index date for a comparison (control) group of patients without DM was the date of a random medical (Dx) or pharmacy (Rx) claim. The non-DM control cohort included patients without a DM diagnosis during the study period *and* with a medical claim ≥ 6 months following a random medical claim between January 1, 2012 and December 31, 2019. Patients in the non-DM control cohort were ≥ 2 year of age at the index date, and patients in the non-cDM control cohort were < 2 year of age at the index date. For both non-DM and non-cDM, no DM diagnosis was recorded during the study period and a 5% random sample (from the database of 249,484,661 claimants) was used to select patients for the analysis who met the aforementioned criteria. A 1:1 propensity score matching technique based on baseline age, gender, index year, payer type, and geographic region, was applied to the DM/ non-DM and cDM/ non-cDM cohorts to reduce bias in control group selection and to ensure a similar distribution of baseline covariates in the two comparison groups [23]. All unmatched control patients were excluded from further analysis. As part of quality control, complete demographic information was required for inclusion in the study.

Comorbidities based on ICD-9/ICD-10 diagnosis codes over the 12-, 24-, 36-, and 48-month follow-up periods for all patients with DM were compared with matched control cohorts. For both disease and control cohorts, each ICD-9 and ICD-10 code was only counted once during a given time period for a single claimant; however, overlapping ICD-9 and ICD-10 codes (due to the introduction of the ICD-10 coding system in October 2015) could be counted as individual instances of a comorbidity in a given time period. In all time periods where the patient associated with a specific ICD-9 or ICD-10 code was present, the comorbidity was recorded (i.e., codes were recorded for an individual in the initial period in which the code was reported and then all subsequent periods in which that individual was present). Thus, the frequency of a given code in any single time period included both new instances and previous instances of that code. Importantly, this overlap does not affect any other measure (e.g., hospitalization), as comorbidities are the only feature of this study presented here that were tabulated as an ongoing event. Comorbidities that affected more than 5% of DM or cDM cohorts and occurred at a ≥ 2 × greater frequency than control cohorts were labelled as “most impactful” for reporting purposes in order to highlight symptom areas where DM differs significantly from controls. This label is not intended to convey impact on quality of life or clinical experience.

HCRU and all-cause costs were analyzed during the first 12 months, 13–23 months, 24–35 months, 36–47 months, and 48 + months for all study cohorts; this included pharmacy, outpatient, and inpatient service categories. For reporting purposes, the 48 + month time period was excluded as it contains all patients remaining at this point in time and is cumulative for as long as their record is active in the database after 48 months of enrolment. As such, the duration could vary for each patient and so it is not comparable to the other, discrete, 12-month time periods. Costs incurred before 2019 were converted to 2019 USD using the medical component of the Consumer Price Index.

Descriptive analyses were generated for all study variables to compare patients with and without DM. Categorical measures were presented as frequency (number of patients, *N*) and percentage (%) of total study patients. Continuous and count variables were reported as the mean, standard deviation (SD), and median. Pre-matched comparisons were conducted for baseline measures using the chi-square (χ^2^) test for categorical variables, and the Wilcoxon rank-sum test (median) and independent sample *t* test (means) were conducted for continuous variables. For matched control cohorts, comparisons were conducted for baseline and follow-up measures using McNemar’s test for categorical variables; the paired *t* test (mean) and Wilcoxon signed-rank test (median) were used for continuous variables. A *P* value of < 0.05 was considered statistically significant. Standardized mean differences (SMD) were reported for the pre- and post-match cohorts. All analyses were based on observed (not projected) data. Outliers were not removed for unadjusted analyses. Analyses were conducted using SAS Release 9.4 (SAS Institute Inc.).

## Results

The pre-matched study cohorts included 8541 patients with DM and 242 patients with cDM and controls (N = 5,595,054 and 344,217, respectively; Table 1). Patients in the DM cohort had a mean (SD) age of 46.0 (18.9) years, which was significantly younger than the pre-matched non-DM control group (48.2 [22.9] years) and had significantly fewer patients in the ≥ 65 years group (16.1% vs. 28.0%). More female patients were present in the DM cohort (55.2%) and also the non-DM cohort (59.6%); however, this was not the case for the cDM (43.4%) and non-cDM (47.5%) cohorts. The majority of DM patients were covered by a third-party insurer (65.5%) as was the pre-matched non-DM control cohort (74.7%), but with significantly greater representation of Medicaid (6.8% DM v. 2.6% non-DM) and Medicare/Medicare Part D (27.6% DM v. 22.7% non-DM) in the affected group. This distribution is similar to that for cDM, with 76.4% covered by a third-party insurer vs. 89.4% for the pre-matched non-cDM control cohort, and significantly greater representation of Medicaid (22.3% DM vs. 8.9% non-DM). Following matching, any differences observed between cohorts based on baseline age, gender, index year, payer type, and geographic region were eliminated (Additional file 1: Table S1). Over the course of the study period, attrition (absence of further claims) occurred for each group, leading to slightly different sample sizes for each cohort within the first 12 months and minor differences in cohort numbers in subsequent time periods.

**Table 1:** Baseline demographic characteristics of patients with DM or cDM compared to pre-matched controls^a^

	DM cohort (*N* = 8541)	Non-DM pre-matched control cohort (*N* = 5,595,054)	*P* value	cDM cohort (*N* = 242)	Non-cDM pre-matched control cohort (*N* = 344,217)	*P* value
*Age, years*						0.1334
Mean ± SD	46.0 ± 18.9	48.2 ± 22.9	< .0001	0.5 ± 0.5	0.5 ± 0.5	
Median	48.0	51.0	< .0001	0	1.0	
*Age group, n (%)*						0.1228
0–11 months				129 (53.3)	166,685 (48.4)	
12–23 months				113 (46.7)	177,532 (51.6)	
2–9 years	353 (4.1)	363,243 (6.5)				
10–17 years	479 (5.6)	353,848 (6.3)				
18–39 years	2003 (23.5)	1,234,691 (22.1)				
≥ 40 years	5706 (66.8)	3,643,272 (65.1)	< .0001			
40–64 years	4328 (50.7)	2,079,171 (37.2)				
≥ 65 years	1378 (16.1)	1,564,101 (28.0)	< .0001			
*Gender, n (%)*						0.2039
Female	4712 (55.2)	3,336,346 (59.6)	< .0001	105 (43.4)	163,394 (47.5)	
Male	3829 (44.8)	2,258,708 (40.4)		137 (56.6)	180,823 (52.5)	
*Payer type*^*b*^*, n (%)*			< .0001			< .0001
Cash	3 (0)	1,214 (0)		0	66 (0)	
Medicaid	585 (6.8)	144,011 (2.6)		54 (22.3)	30,681 (8.9)	
Medicare/medicare part D	2,358 (27.6)	1,269,856 (22.7)		3 (1.2)	5,874 (1.7)	
Third party	5,595 (65.5)	4,179,973 (74.7)		185 (76.4)	307,596 (89.4)	
Other/unknown	0	0		0	0	

DM, myotonic dystrophy; cDM, congenital DM; SD, standard deviation

^a^Assessed on index date if available as first option, or over the pre-index period

^b^Prioritized payer type from a medical claim (Dx) in the pre-index (closest to index date) period or, if none, used payer type from a pharmacy claim (Rx) (closest to index date)

^c^*P* values were obtained using the chi-square test for categorical variables and the Wilcoxon rank-sum test for continuous variables; SMD were used for comparisons between DM cohort vs non-DM control cohort and between cDM cohort vs non-cDM control cohort

### Comorbidities

The study identified over 2000 comorbidities in the DM cohort and over 600 in the cDM cohort. The most impactful comorbidities among patients with DM and cDM, relative to matched controls, are presented in Tables 2 and 3 and Additional file 1: Tables S2 and S3, respectively. During the first 12-month follow-up, patients with DM had significantly increased frequencies of GI system symptoms (14.4% vs 6.6%; ICD-9: 787), cardiac dysrhythmias (12.6% vs 3.6%; ICD-9: 427), and sleep disorders (11.7% vs 3.0%; ICD-9: 327) compared to patients in the non-DM control cohort (*P* < 0.0001 for all). For patients in the cDM cohort, symptoms and signs concerning food and fluid intake (34.7% vs 3.5%; ICD-10: R63), artificial opening status (32.3% vs 0.4%; IDC-10: Z93), and lack of expected normal physiological development (28.0% vs 3.5%; ICD-10: R62) were more prevalent when compared to the non-cDM control cohort (*P* < 0.0001 for all). Among all patients (DM and cDM) and relative to the control cohorts, the number of impactful comorbidities, such as heart failure, malaise and fatigue, and cataracts, increased from the first year to the last year (Additional file 1: Tables S2 and S3).

**Table 2:** Most impactful comorbidities among patients with DM compared with matched controls for the 12-month follow-up period^a^^,b^

ICD-9 or ICD-10 code	Description	DM cohort (*N* = 8,390)		Fold change^c^
		*n*	%	
787	GI system symptoms	1210	14.4	2.2
427	Cardiac dysrhythmias	1054	12.6	3.6
327	Organic sleep disorders	979	11.7	3.9
728	Disorders of muscle/ligament/fascia	957	11.4	3.2
G47	Sleep disorders	934	11.1	2.8
518	Other lung diseases	910	10.8	5.2
R06	Abnormalities of breathing	796	9.5	2.2
M62	Other disorders of muscle	773	9.2	3.4
781	Nervous/musculoskeletal system symptoms	685	8.2	3.9
788	Urinary system symptoms	675	8.0	2.1
794	Abnormal function study	610	7.3	3.6
799	Other ill-defined morbidity/mortality	579	6.9	4.9
785	Cardiovascular system symptoms	568	6.8	2.2
V45^d^	Other postprocedural states	550	6.6	2.2
R53	Malaise and fatigue	686	8.2	2.0

^a^Excludes ICD9 359 (muscular dystrophies) and ICD10 G71 (primary disorders of muscle) as these are the ICD codes for inclusion in the study

^b^Affects more than 5% of the DM Cohort and at least 2X more than the controls with *P* value < 0.0001 during the 12-month follow-up period

^c^Fold change is the multiple of how many more times the disease cohort is affected by the specific comorbidity compared to controls

^d^V45 is the ICD-9 alphanumeric code for “Other postprocedural states”; the same code is also used in ICD-10 to indicate “Car occupant injured in collision with railway train or railway vehicle.” The V45 shown here is an ICD-9 code

**Table 3:** Most impactful comorbidities among patients with cDM compared with matched controls for the 12-month follow-up period^a^^,b^

ICD-9 or ICD-10 code	Description	cDM cohort *(N* = *236)*		Fold change^c^
		*n*	%	
R63	Symptoms and signs concerning food and fluid intake	82	34.7	10.3
Z93	Artificial opening status	76	32.2	76.0
R62	Lack of expected normal physiological development in childhood and adults	66	28.0	8.3
783	Nutrition/metabolism/development symptoms	65	27.5	8.1
R06	Abnormalities of breathing	65	27.5	2.8
R13	Aphagia and dysphagia	62	26.3	62.0
787	GI system symptoms	54	22.9	4.2
J96	Respiratory failure, not elsewhere classifies	49	20.8	24.5
P94	Disorders of muscle tone of newborn	48	20.3	48.0
J98	Other respiratory disorders	47	19.9	11.8
R09	Other symptoms and signs involving the circulatory and respiratory systems	47	19.9	2.9
K21	Gastro-esophageal reflux disease	46	19.5	9.2
R29	Other symptoms and signs involving the nervous and musculoskeletal systems	42	17.8	42.0
Q21	Congenital malformations of cardiac septa	42	17.8	7.0
Z99	Dependence on enabling machines and devices, not elsewhere classified	40	16.9	40.0

^a^Excludes ICD9 359 (muscular dystrophies) and ICD10 G71 (primary disorders of muscle) as these are the ICD codes for inclusion in the study

^b^Affects more than 5% of the DM Cohort and at least 2X more than the controls with *P* value < 0.0001 during the 12-month follow-up period

^c^Fold change is the multiple of how many more times the disease cohort is affected by the specific comorbidity compared to controls

### All-cause healthcare costs

**Fig. 2:**
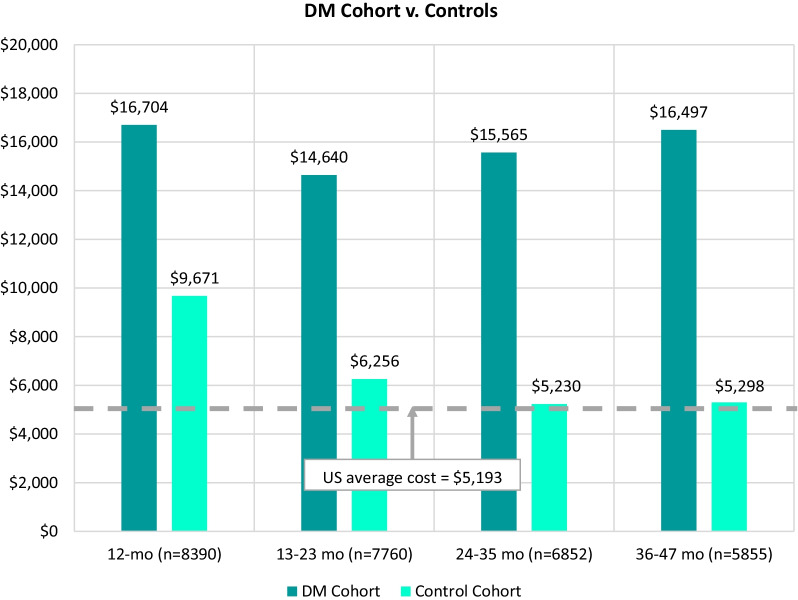
Total annual mean all-cause healthcare cost per patient with DM compared to controls^a^. ^a^Dotted line represents the US average healthcare cost per year as determined by the US Bureau of Labor Statistics [25]

All-cause costs per patient with DM or cDM were compared with matched control cohorts: total pharmacy cost, outpatient cost, physician office visits, ER costs, and inpatient cost per patient were included in the total all-cause costs. The total mean all-cause costs per patient for the DM cohort across the four 12-month follow-up periods compared to the non-DM cohort are shown in Fig. 2. The mean all-cause costs per patient remained high over four years, ranging from between $14,640 and $16,704 and steadily increased from the 13- to 23-month through the 36- to 47-month time periods. In contrast, the control group costs were highest in the first 12 months ($9671) and progressively declined over subsequent time periods, approaching the US population average of $5193. At the 36- to 47-month time period, the mean all-cause costs for the DM cohort ($16,497) were more than 3.7 times higher than that for the non-DM cohort ($5298).

**Fig. 3:**
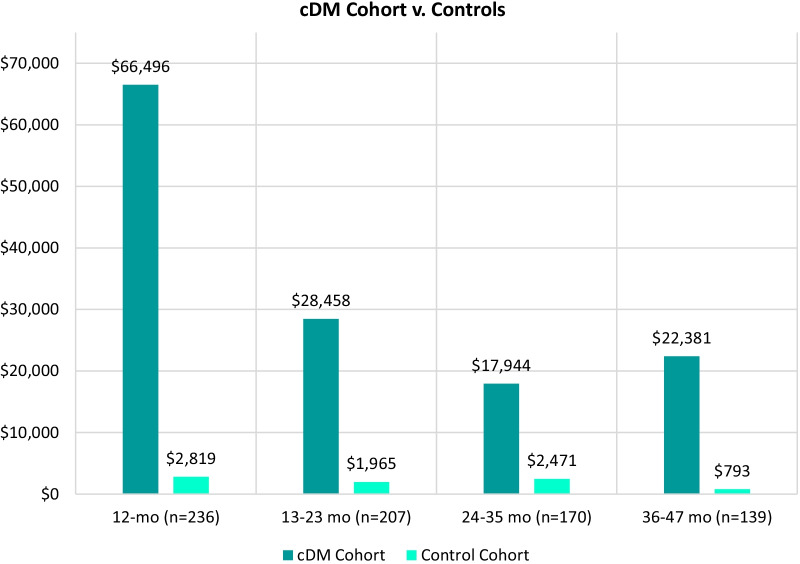
Total annual mean all-cause healthcare cost per patient with cDM compared to controls

For the cDM cohort, the largest difference in comparison to controls was in the first 12-month period, where the cDM cohort mean all-cause costs were $66,496, which is over 23 times that of the non-cDM cohort ($2818; Fig. 3). Over the course of the following time periods, mean all-cause costs for the non-cDM group ranged from $2471 at 24- to 35-months to $793 at 36- to 47-months, while mean costs for the cDM cohort never fell below $17,944. At the 36- to 47-month time period, the mean all-cause costs for cDM were over 28 times higher than that for the non-cDM cohort ($22,381 and $793, respectively).

**Fig. 4:**
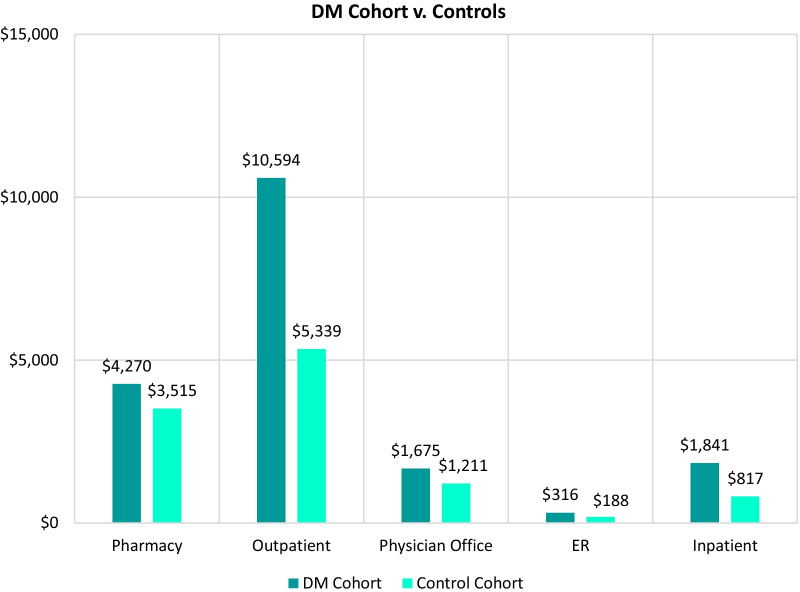
Total annual mean all-cause healthcare cost per patient with DM in the first 12-month study period compared to controls, by cost type

**Fig. 5:**
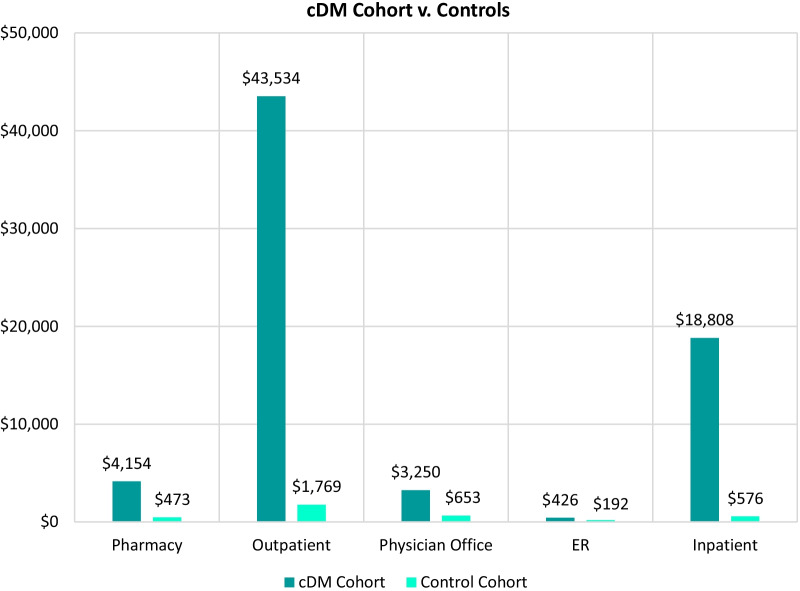
Total annual mean all-cause healthcare cost per patient with cDM in the first 12-month study period compared to controls, by cost type

The mean costs associated with pharmacy, outpatient, physician office visits, ER visits, and inpatient costs are shown for the first 12-month period for DM in Fig. 4 and for cDM in Fig. 5. The second-largest contributing factor to all-cause costs for all cohorts, behind costs for those with ≥ 1 hospitalization, was outpatient costs. Mean outpatient costs for the DM cohort were 1.9 times greater than controls ($10,594 vs $5339) for the 12-month follow-up period and mean outpatient costs for the cDM cohort were 24.6 times greater than controls ($43,534 vs $1769) for the same time period. The mean costs associated with all contributing factors across all study time periods are included in Additional file 1: Tables S4 and S5.

### Total healthcare utilization

Total HCRU was determined for all patients with DM or cDM relative to matched controls (Table 4). During the 12-month follow-up period, patients with DM compared with matched controls had a significantly increased mean (SD) number of prescription fills [34.88 (34.78) vs 29.46 (32.23)], outpatient visits [26.78 (42.40) vs 12.23 (20.35)], physician office visits [8.41 (10.40) vs 6.10 (9.23)], and ER visits [0.73 (2.31) vs 0.45 (1.33)] (*P* < 0.0001 for all). A greater proportion also had ≥ 1 hospitalization (2.9% vs 1.9%, *P* < 0.0001).

**Table 4:** Total HCRU for DM and cDM cohorts compared to matched controls for 12-month Follow-up Period, broken down by resource type

Resource use	DM cohort (n = 8390)			Non-DM control cohort (n = 8297)				cDM cohort (n = 236)			Non-cDM control cohort (n = 228)			
	N (%)	Mean (SD)	Median	N (%)	Mean (SD)	Median	p value	N (%)	Mean (SD)	Median	N (%)	Mean (SD)	Median	p value
*Outpatient pharmacy (in Rx)*														
Patients with ≥ 1 pharmacy record	8388 (100)			8292 (99.9)			0.4142	236 (100)			228 (100)			–
Prescription fills per patient		34.88 (34.78)	24		29.46 (32.28)	19	< .0001		15.45 (17.03)	10		7.30 (7.20)	5	< .0001
*Outpatient medical (in Dx)*														
Patients with ≥ 1 outpatient visit	8381 (99.9)			8258 (99.5)			< .0001	236 (100)			223 (97.8)			< .0001
Number of outpatient visits per patient		26.78 (42.40)	15		12.23 (20.35)	7	< .0001		70.01 (82.77)	42		8.21 (7.13)	7	< .0001
*Physician office visits*														
Patients with ≥ 1 physician office visit	7346 (87.6)			6880 (82.9)			< .0001	196 (83.1)			188 (82.5)			0.8907
Number of physician office visits per patient		8.41 (10.40)	5		6.10 (9.23)	4	< .0001		11.25 (13.95)	7.5		5.86 (5.42)	5	< .0001
*ER visits*														
Patients with ≥ 1 ER visit	2536 (30.2)			1815 (21.9)			< .0001	110 (46.6)			78 (34.2)			0.0080
Number of ER visits per patient		0.73 (2.31)	0		0.45 (1.33)	0	< .0001		1.33 (2.96)	0		0.64 (1.31)	0	0.0003
*Inpatient hospitalizations (In subset with linkage to Charge Description Master)*														
Patients with ≥ 1 hospitalization	247 (2.9)			160 (1.9)			< .0001	18 (7.6)			3 (1.3)			0.0010
Number of hospitalizations per patient		0.05 (0.35)	0		0.03 (0.24)	0	< .0001		0.11 (0.50)	0		0.02 (0.22)	0	0.0032

Similarly, patients with cDM compared with matched control patients had significantly increased mean prescription fills [15.45 (17.03) vs 7.30 (7.20); *P* < 0.0001], physician office visits [11.25 (13.95) vs 5.86 (5.42); *P* < 0.0001], ER visits [1.33 (2.96) vs 0.64 (1.31); *P* = *0.0003*], and a remarkably increased number of outpatient visits [70.01 (82.77) vs 8.21 (7.13); *P* < 0.0001]. A greater proportion also had ≥ 1 hospitalization (7.6% vs. 1.3%, *P* = 0.0010).

The pattern of significant difference in HCRU between DM and non-DM and cDM and non-cDM cohorts is consistent across all four follow-up periods. Additional HCRU in each of the above-listed categories is shown for DM and cDM across all study time periods in Additional file 1: Tables S4 and S5, respectively.

## Discussion

Using claims data derived from the IQVIA New Data Warehouse, this study found that the total all-cause costs and HCRU per patient per year were considerably higher for patients with DM and cDM compared with matched controls. This longitudinal, retrospective, real-world study generated a wealth of data, both in size and number of variables. It is among the first to specifically examine HCRU and total all-cause costs in DM in detail and make efforts to elucidate the experience of those congenitally affected (cDM) [18, 24]. By utilizing a large database and interrogating numerous variables, this study sought to increase understanding of the clinical experience and economic outcomes associated with patients diagnosed with DM, and thereby provide knowledge and support to patients, caregivers, payers as well as those interested in the development of treatments.

The mean all-cause costs per patient per year averaged $15,852 for the DM cohort compared to $6688 for the non-DM cohort. This elevated cost is consistent with the findings of Larkindale and colleagues, who found that direct medical costs per patient with DM per year was $17,451 [18]. For comparison, the US Bureau of Labor Statistics found that the average healthcare cost among people in the US in 2019 was $5193 [25]. For the cDM cohort in this study, the mean all-cause costs per patient per year were $33,820 compared to $2011 for the non-cDM cohort.

Overall costs for the DM cohort are markedly elevated compared to controls across all four study time periods. The relatively high costs for both the DM and non-DM cohorts in the first follow-up period (< 12 months) can be attributed, at least in part, to the increased likelihood that the incident case is associated with an acute, cost-incurring medical event. In subsequent periods however, a trajectory of increasing costs for the DM cohort that reflects the chronic and progressive nature of DM is apparent, while costs for the control group steadily approach the US national average over time.

Total all-cause costs for the cDM cohort compared to their control group follow a similar pattern, in that there is an increase in costs in both the cDM and control group in the < 12 months follow up period; however, for cDM initial costs are extraordinarily high, reflecting the severe nature of cDM and the intensive clinical care interventions required to treat these neonates during this early phase of their lives. In subsequent time periods, the all-cause costs for the cDM cohort decrease, but remain elevated relative to the non-cDM cohort (which decline and approach zero), reflecting the considerable ongoing needs associated with this most severe form of myotonic dystrophy.

Results from this study revealed the following drivers of HRCU among patients with DM or cDM: outpatient, physician office, and ER visits; inpatient hospitalizations; and increased use of prescription medications. For example, the mean number of outpatient visits was 26.78 for the DM cohort vs. 12.23 for controls and the percentage of patients with at least one hospitalization was 2.9% vs. 1.9% in the first 12 months (Table 4) and for every subsequent time point was at least twice that of controls (Additional file 1: Table S4). The observed healthcare utilization patterns align with a previous study that examined hospital system claims data and patient records for DM patients in that both studies demonstrate increased HCRU: study authors found that 55.0% of patients with DM had at least one physician office visit, 64.2% had one ER visit, and 44.6% had at least one inpatient visit over the course of the 5-year study period [24]. While the rate of inpatient visits reported (44.6%) is higher than the 2.9% reported here, this increased frequency of hospitalization is due to study design differences—the study by Bennett et al. utilized hospital databases exclusively and reported cumulative frequencies for the entire study period.

In this study, numerous comorbidities were identified at varying frequencies and levels of significant difference compared to controls that illustrate the disease burden in DM. The detailed comorbidity findings are not presented here but the data tables are available for further analysis upon request. The most impactful comorbidities (defined above) varied with DM type and also highlighted underappreciated symptoms, which not only can dramatically affect quality of life, but can interfere with functions important in work and/or academic performance (e.g., sleep disorders). It should also be noted that the comorbidities identified in this study negatively affected the patient to a degree that prompted the patient to present to a medical facility for care or came to the attention of the treating clinician. During the first follow-up year, GI system symptoms (14.4%), cardiac dysrhythmias (12.6%), and organic sleep disorders (11.7%) were the most prevalent comorbidities among patients in the DM cohort (Table 2); however, for the cDM cohort, symptoms and signs concerning food and fluid intake (34.7%), artificial opening status (32.2%) and development (28.0%) were most prevalent (Table 3). Consistent with previously identified comorbidities for DM, pneumonia, respiratory failure, and cardiac arrhythmia, were found to be 5.5%, 5.1%, and 3.0%, respectively, for the DM cohort during the study period [1, 16, 26]. Moreover, identified comorbidities, including sleep disorders, cataract, fatigue, and dysphagia are consistent with the multi-systemic nature of DM as previously described [1, 2, 16, 26, 27]. Furthermore, patients with cDM had comorbidities including respiratory disorders, lung diseases, cardiac abnormalities, and hearing loss that are consistent with previous research findings [26, 28]. Importantly, due to the transition from ICD-9 to ICD-10 mid-study (October 2015) the reported frequency counts for any individual comorbidity (ICD-9 *or* ICD-10 code) represent a conservative estimate of true incidence/prevalence. For the purposes of analysis, ICD-9 codes were not combined with corresponding/equivalent ICD-10 codes to avoid double counting codes for cases that crossed the code transition date, which would result in an overestimation. While the relative frequency (compared to controls) reported for any given ICD-9 or ICD-10 code is reliable, the absolute frequency is likely an underestimate.

Overall, the significantly higher all-cause costs and increased HCRU reflect the elevated comorbidity profiles and varying needs among patients with DM and cDM, in terms of both disease management and for the payer. Furthermore, as the follow-up duration of the study progressed, the number of comorbidities among the DM and cDM cohorts increased and the annual costs remained significantly higher relative to the control cohorts, consistent with the chronic and slowly-progressive nature of DM.

This study has limitations inherent to a longitudinal and retrospective analysis using a large insurance claims database. First, in an open claims database, there are no true enrollment start and stop dates and thus data capture may underestimate actual services used. In addition, there was no differentiation between DM1 and DM2, since the ICD-9/ICD-10 code for DM does not distinguish between the two subtypes. While the use of claims data is valuable for evaluating HCRU patterns, total costs, and comorbidities, they are primarily used for billing purposes; this inherently affects their generalization to the public, as they can only be applied to the insured population. Within the dataset, the application of the DM diagnosis code may be inconsistent or sporadic, and its presence does not necessarily indicate disease and vice versa. This risk was mitigated by requiring patients have at least two DM diagnosis codes, but still may have influenced the study results. This rigor may have resulted in a study population that does not capture the full spectrum of the DM patient journey. It may not capture DM and cDM who had a short interval between diagnosis and death, or patients who do not seek regular care. It should also be noted that the costs reported here are the amounts of money the payer reimbursed the individual or institution, and do not include expenses absorbed by individual patients, families, or the institutions that serve them. Additionally, these claims data include only those services for which the insurers will pay, and may exclude important and necessary items like uninsured aids and assistive devices, home healthcare, other non-insured expenses, and/or loss of work or income due to illness [18]. This dataset also does not identify those DM and cDM for patients who have not yet received a diagnosis, nor those that have declined genetic testing for fear of losing their insurance or other potential negative outcomes.

## Conclusions

This retrospective study found that patients with congenital and non-congenital DM had elevated comorbidity profiles. In turn, these patients had higher HCRU each year following initial diagnostic coding of DM or cDM compared with matched cohorts of patients without DM. Therefore, the DM and cDM study cohorts were found to have higher total annual healthcare costs during each 12-month follow-up period after diagnosis compared with matched control patients. The wide array of impactful comorbidities identified in this study highlights the need for collaborative care as well as for treatments that relieve the symptomatic burden of disease. Together, the markedly elevated HCRU and comorbidity profile presented here, along with the broad body of scientific knowledge on DM and cDM, can be used to support development of disease modifying and/or symptom-targeting therapies that adequately address the multi-systemic nature of DM. Given the chronic and progressive nature of the disease and the absence of approved treatments or medications, the need is an urgent one.

### Further research/future considerations

This study is among the first to establish a baseline understanding of the clinical and economic outcomes associated with DM, thereby providing a benefit to patients, caregivers, and payers. The findings provide key insights into HCRU and healthcare costs in this understudied patient population and contribute to the understanding of disease burden prior to approved therapies. Future work to examine the association of DM severity and higher HCRU and cost may provide additional insight into the predictive factors for increased economic burden.

## Supplementary Information

**Additional file 1**. Additional information including post-match demographic data, most impactful comorbidities, total HCRU, and total all-cause cost data for DM and cDM groups and controls for all study periods is provided in Supplementary Tables S1-S5 (in excel format).** Table S1**. Baseline Demographic Characteristics (post-match).** Table S2**. DM Cohort Most Impactful Comorbidities.** Table S3**. cDM Cohort Most Impactful Comorbidities.** Table S4**. Total HCRU & All-cause Costs for DM Cohort over 12-, 13-23, 24-35, 36-47 & 48+ Month Follow-Up Periods.** Table S5**. Total HCRU & All-cause Costs for cDM Cohort over 12-, 13-23, 24-35, 36-47 & 48+ Month Follow-Up Periods.

## Data Availability

The data tables from the current study are available from the corresponding author on reasonable request.
