# Correction: Healthcare resource utilization, total costs, and comorbidities among patients with myotonic dystrophy using U.S. insurance claims data from 2012 to 2019

**DOI:** 10.1186/s13023-022-02401-x

**Published:** 2022-07-11

**Authors:** Sarah J. Howe, David Lapidus, Michael Hull, Jason Yeaw, Tanya Stevenson, Jacinda B. Sampson

**Affiliations:** 1 Marigold Foundation, 7515 Flint Road SE, Calgary, AB T2H 1G3 Canada; 2 LapidusData Inc., Oklahoma City, OK USA; 3 IQVIA, Falls Church, VA USA; 4 Myotonic Dystrophy Foundation, Oakland, CA USA; 5 Stanford University, Stanford, CA USA

## Correction to: Orphanet Journal of Rare Diseases (2022) 17:79 10.1186/s13023-022-02241-9

Following the publication of the original article [1] the authors informed us that the second author’s surname “Ladipus” should be corrected to “Lapidus”.

The correct author name, "David Lapidus", is included in the author list of this Correction, and has already been updated in the original article.
